# The Role of IgA in Chronic Upper Airway Disease: Friend or Foe?

**DOI:** 10.3389/falgy.2022.852546

**Published:** 2022-03-09

**Authors:** Alba Sánchez Montalvo, Sophie Gohy, Philippe Rombaux, Charles Pilette, Valérie Hox

**Affiliations:** ^1^Pole of Pneumology, ENT and Dermatology, Institute of Experimental and Clinical Research, Université Catholique de Louvain (UCLouvain), Brussels, Belgium; ^2^Allergy and Clinical Immunology Research Group, Department of Microbiology, Immunology and Transplantation, KU Leuven, Leuven, Belgium; ^3^Department of Pulmonology, Cliniques Universitaires Saint-Luc, Brussels, Belgium; ^4^Cystic Fibrosis Reference Center, Cliniques Universitaires Saint-Luc, Brussels, Belgium; ^5^Department of Otorhinolaryngology, Cliniques Universitaires Saint-Luc, Brussels, Belgium

**Keywords:** rhinitis, rhinosinusitis, allergy, polyps, epithelium, mucosal immunity, IgA

## Abstract

Chronic upper airway inflammation is amongst the most prevalent chronic disease entities in the Western world with prevalence around 30% (rhinitis) and 11% (rhinosinusitis). Chronic rhinitis and rhinosinusitis may severely impair the quality of life, leading to a significant socio-economic burden. It becomes more and more clear that the respiratory mucosa which forms a physiological as well as chemical barrier for inhaled particles, plays a key role in maintaining homeostasis and driving disease. In a healthy state, the mucosal immune system provides protection against pathogens as well as maintains a tolerance toward non-harmful commensal microbes and benign environmental substances such as allergens. One of the most important players of the mucosal immune system is immunoglobulin (Ig) A, which is well-studied in gut research where it has emerged as a key factor in creating tolerance to potential food allergens and maintaining a healthy microbiome. Although, it is very likely that IgA plays a similar role at the level of the respiratory epithelium, very little research has been performed on the role of this protein in the airways, especially in chronic upper airway diseases. This review summarizes what is known about IgA in upper airway homeostasis, as well as in rhinitis and rhinosinusitis, including current and possible new treatments that may interfere with the IgA system. By doing so, we identify unmet needs in exploring the different roles of IgA in the upper airways required to find new biomarkers or therapeutic options for treating chronic rhinitis and rhinosinusitis.

## Introduction

Chronic inflammatory upper airway diseases are among the most prevalent chronic disease entities impacting the life of about 25% of the population in the Western World (1, 2). The most common presentation form of upper airway disease is rhinitis, which is defined as an inflammation of nasal mucosa, leading to symptoms of nasal blockage, rhinorrhea, sneezing and itch (3). Acute rhinitis or common cold has mostly an infectious origin, while the most frequent cause of chronic rhinitis is IgE-mediated allergic inflammation. When the inflammation extends to the mucosa of the paranasal sinuses, it is addressed as rhinosinusitis, which has been shown to affect about 11% of the European population (4). Chronic rhinosinusitis (CRS) is a very heterogeneous disease that can present either with or without nasal polyposis (CRSwNP or CRSsNP, respectively). Recently, due to the development of novel biotherapeutics options, CRS is being classified according to its inflammatory subtype (5, 6). The majority of Caucasian CRSwNP patients present with an eosinophilic type 2 inflammatory subtype, as well as 50% of the CRSsNP population (2). Type 2 inflammation is characterized by the presence of Th2 and ILC2 cells and the secretion of IL-4, IL-5, and IL-13 leading to the accumulation of eosinophils and mucus production. In this type 2 patient group, a substantial proportion of patients (especially those with severe disease) are colonized with *Staphylococcus aureus* and show a polyclonal IgE response toward its enterotoxins (7). Yet, still a lot of open questions remain regarding its pathophysiology and disease modulation. The remaining half of CRSsNP patients, as well as a minority of CRSwNP patients, are characterized by a non-type 2 inflammation (2). This consists of a mix of Th1, Th17, and Th22 cells, in the absence of IgE (2), leading to the secretion of IL-6, IL-8, TNFα, and IFNγ resulting in neutrophilic inflammation. The disease drivers and molecular mechanisms underlying this non-type 2 CRS are even less understood than NP disease.

Currently, patients are frequently managed by corticosteroids, antibiotics or, more recently, biologicals. Despite these treatments, still a substantial number of patients remains poorly controlled (8) and more insight in disease mechanisms is needed to develop novel therapeutic options, with less side-effects.

Although some light has been shed on the pathophysiology of rhinitis and rhinosinusitis in the past decade, still a lot of question marks remain regarding disease drivers and modifiers of chronic inflammatory upper airway diseases. The nose and sinuses are situated at the entrance of the airways, and therefore encounter a plethora of inhaled agents each day, which need to be dealt with by the nasal mucosal lining and its immune system. In case of failure of this mucosal barrier to maintain tolerogenic immune responses against environmental or microbiota-derived antigens, chronic inflammation can arise leading to the above-mentioned pathologies. Within the humoral immune system, immunoglobulin E (IgE) receives most of the attention because of its well-studied role in allergic rhinitis (AR) (3) and in CRS with NP (CRSwNP) (9), however, the potential contribution of other immunoglobulins in rhinitis and rhinosinusitis has been very little studied. Interestingly, immunoglobulin A (IgA) is the most abundant immunoglobulin in nasal secretions where it is considered an innate immunoprotein that contributes to the first-line immune defense toward inhaled antigens (10). Data on the role of IgA and its receptors in the upper airways is partly coming from individuals presenting with selective IgA deficiency (SIgAD). SIgAD is defined as decreased or absent levels of IgA in the serum (generally <7 mg/dl) with normal levels of IgG and IgM. It is the most common immune deficiency worldwide estimated to affect around 0.7% of the Caucasian population (4). In addition to these low serum levels, it has been demonstrated that these patients, show absent or very low production of IgA at the level of their upper airways (11). Initially, the role of IgA in the upper airways was considered passive, since these individuals do not present with an overt infectious or inflammatory phenotype. However, recent research suggests a more active role of IgA in immunity (12), and disturbances in IgA biology have been linked to the pathophysiology of rhinitis (13, 14) and chronic rhinosinusitis (15).

In this review, we summarize what is currently known in literature on the role of IgA in upper airway homeostasis as well as disease. We discuss how current treatments for upper airway pathology might interfere with IgA biology and which novel opportunities might be explored to make upper airway disease patients benefit from this immunoprotein.

## IgA Biology

IgA was first described in 1953 and is the most abundant immunoglobulin produced in external fluids (16). Human IgA comprises two heavy and two light chains and it occurs in 2 isotype forms, IgA1 and IgA2. Serum IgA, which predominantly consists of monomeric A1 (80%), is produced in the bone marrow, while in secretory IgA there is an increased proportion of IgA2, the subtype that is relatively more resistant to enzymatic degradation (17).

Serum IgA is largely present as a monomer in humans and it can bind to various receptors expressed by granulocytes, monocytes, macrophages, dendritic cells (DC), and eosinophils, including the myeloid-cell-specific type I Fc receptor for IgA (FcαRI or CD89), the Fcα/Fcμ receptor, the asialoglycoprotein receptor, and the transferrin receptor (18). The effector functions of these receptors remain poorly understood and involve both pro-inflammatory as well anti-inflammatory pathways. Anti-inflammatory signals are generated by FcαRI upon binding of monomeric IgA, whereas pro-inflammatory FcαRI-dependent responses are induced by IgA immune complexes (19).

Although IgA is present in the serum, its main known functions are elicited at the mucosal level under the form of secretory IgA (S-IgA) which is almost entirely produced locally by plasma cells. The luminal epithelium is continuously exposed to exogeneous antigens, which are taken up by subepithelial antigen presenting cells (APC). These APCs then process the antigens and induce mucosal immune responses in local lymphoepithelial structures which are generally addressed as mucosa-associated lymphoid tissue (MALT). At the mucosal barrier of the upper airways, this MALT is contained in the nasopharynx, across the nasal epithelium and at the level of the tonsils and adenoids (Waldeyer's ring), which is addressed as the nasal-associated lymphoid tissue (NALT) (20). Generally, in MALT, naïve B-cells are primed by T-cells that have been activated by APCs that have processed their luminal antigen. In response to this activation, B-cells in the follicles will undergo somatic hypermutation leading to the development of memory cells and the production of antigen-specific antibodies, among which IgA. IgA production typically occurs in response to transforming growth factor-β1 (TGFβ1), that activates the specific promoters responsible for IgA class switching. This makes TGFβ1 essential for the induction of T-cell-dependent IgA class switching. In addition to TGFβ1, it has been described that IgA switching can also occur through B-cell activation in combination with other cytokines, such as interleukin-2 (IL-2), IL-4, IL-5, IL-6, and IL-10 (20–26). The MALT provides a unique environment that promotes the development of IgA-producing B cells (27), meaning that primed B-cells in the MALT system that have encounter their cognate antigen, mature into conventional B2 cells that produce antigen-specific IgA (28, 29). These cells will then home to the sinonasal epithelium where they will differentiate into IgA-secreting plasma cells and provide a specific defense against harmful antigens.

On the other hand, to be able to provide an immediate IgA response to inhaled antigens, B-cells can also be instructed to produce non-specific, polyreactive IgA in a T-cell independent way. Certain T-cell independent antigens do this by directly activating B cells; e.g., lipopolysaccharide (LPS) *via* Toll-like receptors (TLRs) (30) or polysaccharides through the BCR (31). Also, retinoic acid has been shown to induce a selective IgA class switch in B cells (32). Other T-cell-independent antigens induce an IgA class switch in an indirect way, *via* promoting the production of B-cell activating factor (BAFF), and a proliferation-inducing ligand (APRIL) in DCs that lead to the B-cell production of IgA1 and IgA2, respectively (33, 34). But also epithelial cells and local stromal cells may contribute to T cell-independent production of local IgA by secreting thymic stromal lymphopoietin (TSLP), IL-6, IL-10, BAFF, and APRIL (35). TACI (transmembrane activator and CAML interactor), a transmembrane receptor of the TNF receptor family that is expressed on B-cells, binds BAFF and APRIL and this interaction plays a major role in the class switch to IgA. The B-cells that have been activated *via* this T-cell independent system, are more of an innate type B-cell (B1 cells) and they have been described in the gut to secrete unmutated or “natural” IgA. This natural IgA shows spontaneous antigenic specificities to naturally occurring epitopes at the surface of erythrocytes, thymocytes and microorganisms such as phosphorylcholine or LPS (35). These polyreactive antibodies recognize multiple antigens with low affinity and are therefore able to provide limited protection against a plethora of pathogens (36–38).

**Figure 1:**
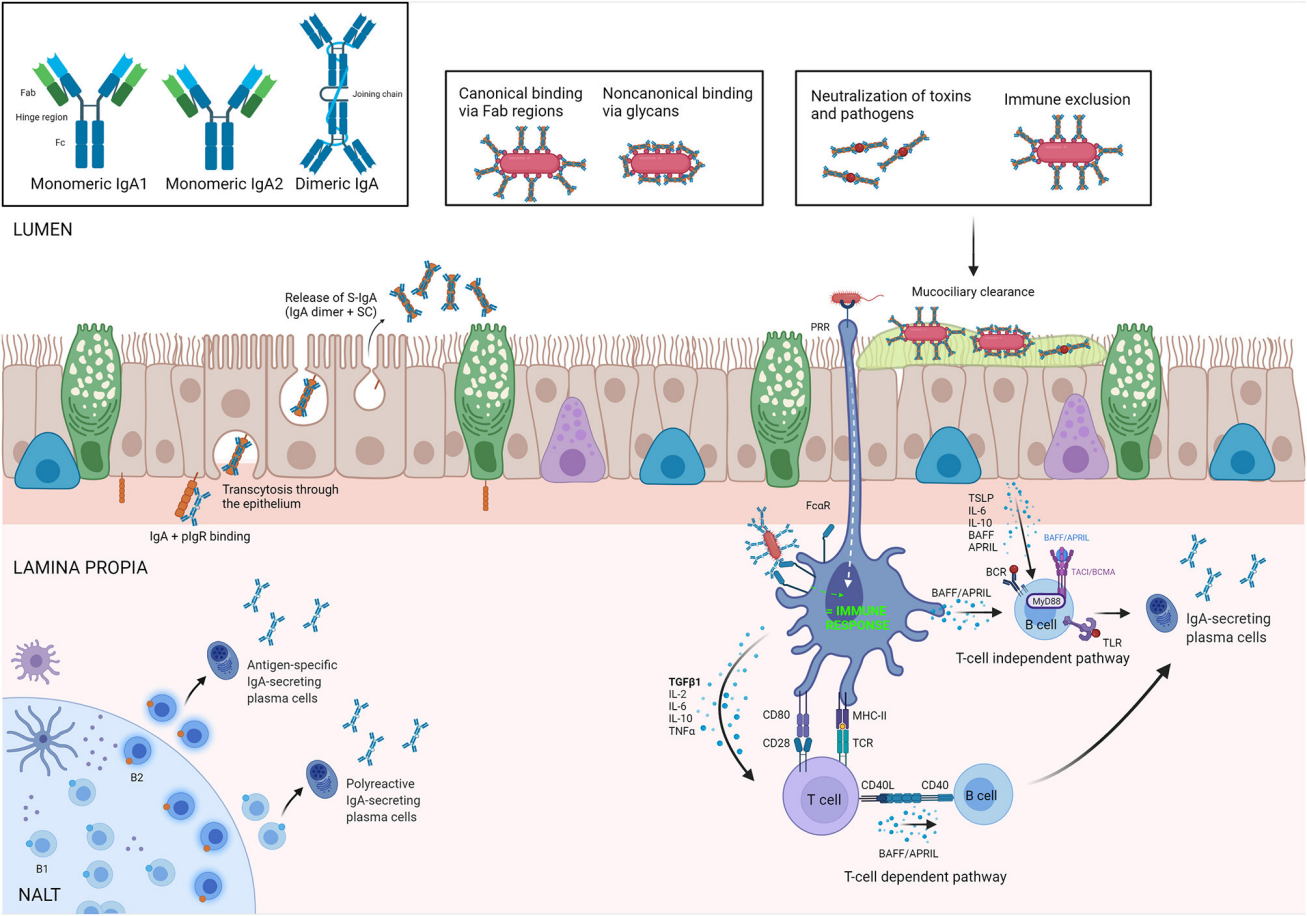
IgA biology. Isoforms of IgA are illustrated in upper-left square. In the Nasal Asocciated Lymphoid Tissue (NALT), antigen-specific and polyreactive IgA is produced by B2 and B1 cells, respectively, and secreted in the lamina propria by plasma cells as dimeric IgA (dIgA). The polymeric immunoglobulin receptor (pIgR) is responsible for its transepithelial transport toward the lumen, where dIgA is released bound to the secretory component (SC). The complex IgA/SC is what we know as secretory IgA (S-IgA). In the lumen, S-IgA binds toxins and allergens in either a canonical and/or non-canonical way to perform its main roles: neutralization and immune exclusion, preventing bacterial colonization by invasive species (panel inserts). Antigen-activated T-cells induce an IgA class switch in B-cells, typically in the presence of Transforming Growth Factor beta (TGF-β), or other mediators secreted by dendritic cells (DC) or epithelium. T-cell independent IgA class switch occurs either *via* direct antigenic activation of B-cells or *via* production of B cell activation factor (BAFF) and/or A proliferation inducing ligand (APRIL) by DCs. TSLP, thymic stromal lymphopoietin; PRR, pattern-recognition receptor; TLR, toll like receptor; TACI, transmembrane activator and calcium modulating cyclophilin ligand interactor. Created with BioRender.com.

After secretion by plasma cells homed to the lamina propria, IgA forms dimers comprising two IgA monomers that are covalently linked to a polypeptide known as the J-chain (39). The J-chain of the dimeric IgA (d-IgA) can bind to the polymeric immunoglobulin receptor (pIgR) that is expressed at the basolateral site of nasal epithelial cells and the d-IgA/pIgR complex is then transcytosed toward the mucosal lumen. Once the apical site of the epithelium is reached, a proteolytic cleavage occurs and d-IgA is released, bound to the main and heavily glycosylated part of the extracellular domain of pIgR, known as secretory component (SC), to form secretory IgA (S-IgA). SC protects the complex from proteolysis. In the gut, it has been shown that S-IgA binds to the mucus layer covering the epithelial cells, reinforcing the mucosal barrier, making it capable of neutralizing threats before they reach the epithelial cells. It also mediates the transepithelial transport of the bound antigen from the intestinal lumen to the MALT where it is then internalized by DCs in the subepithelial region (40). Figure 1 summarizes the most important pathways of the IgA/pIgR system.

## Role of IgA in Upper Airway Homeostasis

**Figure 2:**
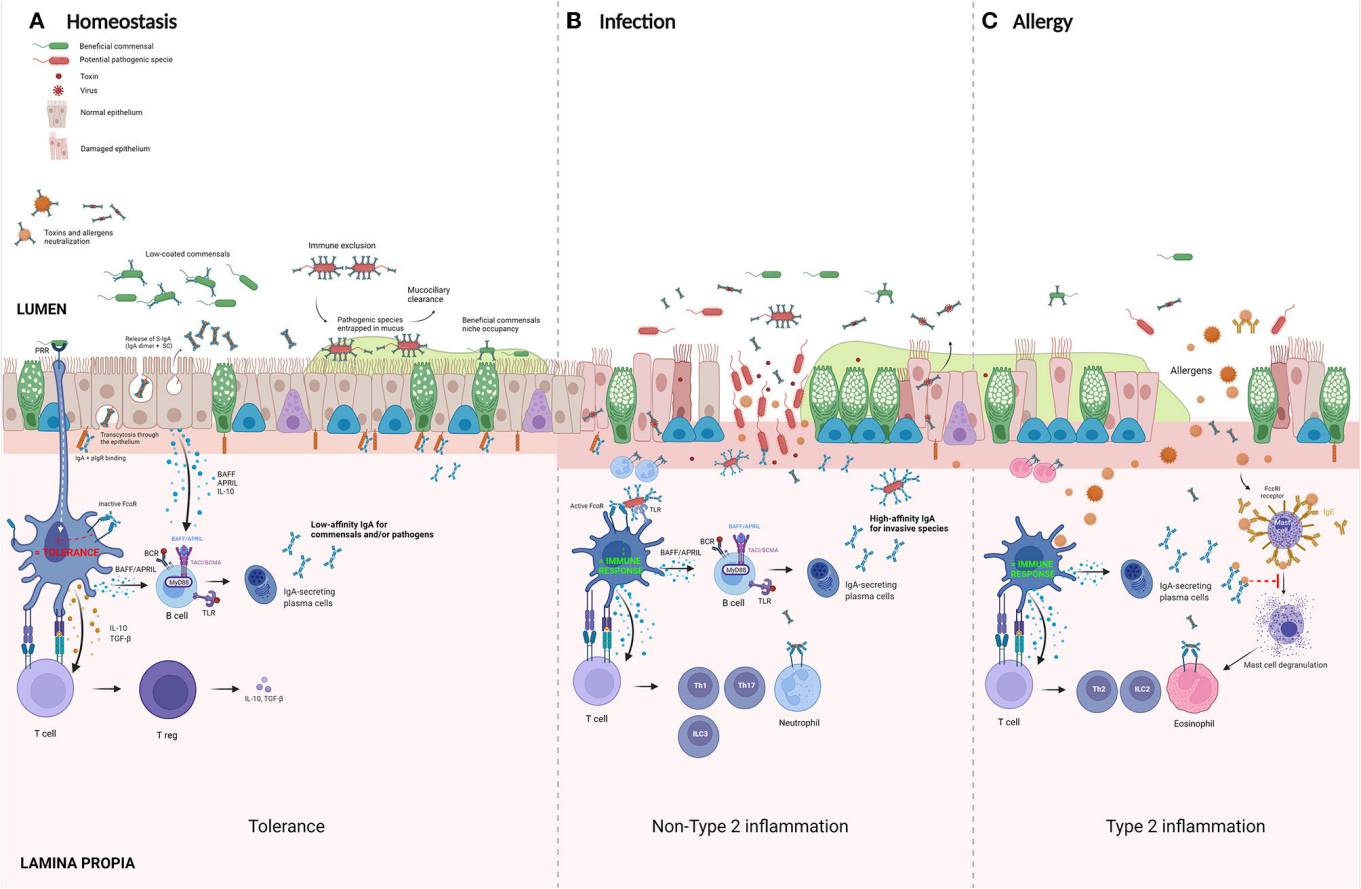
Suggested roles of IgA in homeostasis and disease. **(A)** Homeostasis panel: commensal bacteria colonize mucosal surfaces of the respiratory tract. IgA-secreting plasma cells produce IgA that binds microbes with low affinity, preventing exclusion of coated bacteria. In this way, IgA coating controls the composition of the microbiome by promoting growth of beneficial commensals. Isolated activation of FcαR on dendritic cells (DC) induces tolerance *via* the activation of T regulatory cells. **(B)** Infection panel: pathogens that penetrate the epithelial layer will be opsonized by local dimeric IgA (dIgA) present in the subepithelial space. These antibody-opsonized pathogens will activate DC by a double stimulation of both FcαRI and pathogen-recognition receptors (PRR), such as Toll-like receptors (TLRs). This costimulation turns tolerogenic DCs into pro-inflammatory cells which induce an active adaptive immune response by activating T helper (Th) 1, Th17, and type 3 innate lymphoid cells (ILC). **(C)** Allergy panel: in atopic individuals, the uptake of allergens by DC results in an induction of a type 2 inflammatory environment with the induction of Th2 cells and activation of ILC2 with an influx of eosinophils and production of antigen-specific IgE. In these sensitized individuals, re-exposure to allergens causes a cross-linking of mast-cell bound IgE on mast cells, leading to their degranulation. Secretory IgA (S-IgA) is known to bind and activate eosinophils and might promote this Th2 response. However, antigen-specific IgA is believed to act as a scavenger for allergen, preventing binding to its specific IgE. In the pre-sensitization phase, antigen-specific S-IgA is believed to play an important role in inducing allergenic tolerance. BAFF, B cell activation factor; APRIL, A proliferation inducing ligand; TSLP, thymic stromal lymphopoietin; PRR, pattern-recognition receptor; IgA-IC, IgA-immune complexes. Created with BioRender.com.

In contrast to most other antibodies, IgA has both immunosuppressive and pro-inflammatory characteristics. Because of this feature, IgA is one of the most important molecules in maintaining a homeostasis at the mucosal barrier, which is permanently exposed to an enormous variety of both harmful and harmless (even beneficial) antigens. In the gut, IgA has shown to promote mucosal homeostasis in three different ways: [1] by neutralizing potentially hazardous microbes, [2] by shaping the composition of the commensal microbiota, and [3] by preventing inappropriate inflammatory responses to microbial and food antigens. Although, fewer data is available on these functions of IgA in the airways, similar mechanisms might be playing in respiratory mucosal homeostasis. Figure 2 summarizes the most important pathways of IgA in both homeostasis and bacterial/allergen-induced inflammation.

### Induction of Mucosal Tolerance Against Harmless Antigens

Besides its role of a neutralizing antibody that excludes antigens, S-IgA has also a function in antigen sampling, allowing a selective transcytosis of antigens from the lumen to the MALT (40). Not all encountered antigens form a threat for the host and it is essential that not all antigens lead to an activation of the immune system in order to avoid the development of aberrant inflammatory responses. Ideally, this means that upon inhalation of a harmless antigen, a suppression or down-regulation of the immune effector cell responses is induced and this immune suppressive effect is addressed as mucosal tolerance.

S-IgA is a key player in maintaining this mucosal tolerance, mainly because of is its inability to trigger an inflammatory response after binding its specific receptor FcαRI individually. FcαRI, which is expressed by the myeloid cell compartment, needs to collaborate with other receptors as mentioned above in order to amplify or inhibit the production of certain cytokines. The binding of IgA to its antigen leads to the formation of immune complexes (ICs) and their capacity to induce either tolerance or inflammation *via* the FcαRI depends on the absence or presence of a second signal. In the absence of additional signals, the recognition of ICs by FcαRI facilitates the uptake of antigens which leads to only a partial activation of DCs and does not trigger any proinflammatory responses (41). This function is key in the induction of the development of mucosal tolerance to, e.g., certain inhaled allergens where S-IgA seems to play a role (see below), although still very little is known about its mechanisms.

### Protection Against Pathogenic Infections

In addition to maintaining the mucosal tolerance, S-IgA plays an important role in the first-line immune defense against harmful inhaled agents such as pathogenic microbiota. It is able to provide this defense function through different mechanisms at different locations within the mucosa.

IgA can bind microbes in both a canonical and non-canonical way (42). Canonical binding includes Fab region-dependent binding of the antibody to the microbial antigen, while non-canonical binding describes all other binding modalities that are not determined by the complementarity determining regions (CDR) at the end of the Fab arms.

One of the oldest functions attributed to S-IgA is immune exclusion. Immune exclusion comprises of a series of events being agglutination, mucus entrapment and clearance of the airways (43). S-IgA first blocks the adherence of toxins and bacterial surface antigens, such as adhesins or pili that promote epithelial invasion by direct recognition of receptor binding domains (44). Then, agglutination occurs *via* crosslinking of IgA with microbial antigens, leading to the formation of macroscopic clumps of pathogens. It has been shown in both the gut as well as in the airways that those agglutinated clumps are then entrapped in the epithelial mucus layer which leads to their clearance out of the airways (45, 46). This entrapment is mostly mediated thanks to the oligosaccharide side chains of SC (47, 48) which emphasizes the advantages of S-IgA over monomeric IgA in secretions. At the level of the nasal mucosa, it has been shown that Fab-dependent recognition of S-IgA of a specific *Streptococcus pneumoniae* pilus protein, was required for binding to nasal fluid, leading to bacterial agglutination and immune exclusion. Also in mice, S-IgA treatment resulted in rapid immune exclusion of pilus-expressing pneumococci from the airways (49). This S-IgA function is addressed as passive immunity because it is able to eliminate pathogens from the lumen without the initiation of an adaptive immune response. In addition to eliciting this passive immune function in the lumen, specific IgA antibodies have also been shown to neutralize intracellular viruses (50) or proinflammatory antigens such as LPS (51) while in transit through the epithelium.

Nevertheless, although the rather poor complement activation functions of IgA in comparison to IgG and IgM, subepithelial dimeric (d) IgA is also able to activate an adaptive immune response. Pathogens that penetrate the epithelial layer will be opsonized by local d-IgA present in the subepithelial space. Under homeostatic conditions, IgA-opsonized pathogens are scarce in the lamina propria, but become very high on invasion by microorganisms during infection. These antibody-opsonized pathogens will not only activate FcαRI but also pathogen-sensing receptors, such as Toll-like receptors (TLRs), providing a second signal to the same immune cell. The combination of these two signals can then co-activate otherwise tolerogenic DCs into pro-inflammatory cells which induce an active adaptive immune response by activating T helper (Th)17 cells and type 3 innate lymphoid cell (ILC) responses (52).

The importance of IgA in the protective anti-bacterial immune response seems clear, since bacteria evolved into developing anti-IgA or anti-FcαRI bacterial proteins that prevent IgA-FcαRI interactions in order to avoid phagocytosis by FcαRI-expressing cells (53).

### IgA and Microbiome Homeostasis

It is known that the nose and paranasal sinuses house a diverse microbiome with both commensal and pathogenic bacteria (54, 55). In healthy individuals, commensal microbes form a symbiosis with the host and they can even contribute to the mucosal barrier against incoming external pathogens. One of the most important functions of the mucosal immune system is to distinguish between pathogenic agents and innocuous commensals.

Because both commensals and invading pathogens often express similar antigens, the recognition of microbial structures by pathogen recognition receptors (PRR) such as TLRs is insufficient to explain how the mucosal immune system can discriminate commensals from invading bacteria (52). In view of the immunomodulatory capacities of IgA, it is believed that this antibody plays a crucial role in this distinction which has been investigated largely in the gut. Here, it has been reported that IgA might be responsible for the maintenance rather than elimination of indigenous bacteria to keep the diversity in gut (56). At the level of the intestinal mucosa, it is known that IgA binds to the surface of certain members of the intestinal microbiota (it has been estimated that up to 74% of bacteria in the gut lumen are coated with S-IgA) and for the majority of these microbes, this does not lead to their clearance from the intestinal system (57). Recent publications have revealed new properties of this IgA coating, including the control of the host's tolerance toward certain commensals (58). Why certain microbes are IgA-coated and others not, still remains a question mark. It has been suggested that IgA can directly or indirectly modulate the gene expression of microbes (59, 60) leading to different responses from epithelial or other cells toward the bacteria depending on their IgA-coating (60). Another explanation might be that bacterial IgA-coating depends on and might even control the pathogenicity of the microbe; Palm and colleagues combined both human and murine samples of chronic inflammatory bowel disease and found that IgA-coating of microbiota determined the “colitogenic” capacity of the concerned bacteria (61). More prove to support this theory is coming from patients suffering from SIgAD that seem to present with a dysbiosis of the gut microbiota, characterized by overrepresentation of certain pathogenic species, as well as a simultaneous underrepresentation of certain beneficial commensals (62–64). It has also been shown that S-IgA coating of micro-organisms can facilitate the formation of a biofilm that favors the growth of slow-growing commensals while attenuating that of pathogens (65).

There is some minor evidence that similar pathways may be playing in the airways. For example, pIgR^−/−^ mice that completely lack S-IgA, show a persistent activation of innate immune responses to resident lung microbiota, driving progressive small airway remodeling and emphysema (66). Interestingly, it has recently also been shown that disrupted SIgA-microbiota interactions in the gut are linked to an increased risk of developing allergies and asthma in children (67). This does not only emphasize the importance of IgA-microbial interactions in the development of allergies, but also the potential systemic signaling and interchangeability of the different microbial compartments in the human body.

## Role of IgA in Upper Airway Disease

Unlike gastro-intestinal diseases, the role of IgA in airway disease remains largely understudied. Up till now, some articles have reported on its function in chronic obstructive pulmonary disease (COPD) (68), asthma (68), and cystic fibrosis (69). Data on the role of IgA and its receptors in upper airway disease are even more scarce and mostly linked to what is known from patients presenting with SIgAD. Despite the seemingly critical roles of S-IgA, the majority of patients diagnosed with SIgAD are asymptomatic. This might be explained by either inadequate diagnostic testing or compensation mechanisms by other immunoglobulins. Nevertheless, these patients seem to present with more frequent respiratory infections, allergies, and auto-immune diseases compared to patients with normal IgA levels (70, 71). Below, we list what is known about the contribution of IgA and its receptor to the different types of upper airway dysfunction.

### Role of IgA in Recurrent Upper Airway Infections

Viral rhinitis or common cold is one of the most common conditions in the world. In case of bacterial superinfection, rhinitis will virtually always evolve to a bacterial rhinosinusitis.

Due to the important role of IgA in first line defense against inhaled pathogens, leading to their neutralization and elimination out of the respiratory tract, people presenting with SIgAD are expected to suffer from recurrent airway infections. Indeed, epidemiological studies haves shown that recurrent infections of the respiratory system are the most common finding in patients with SIgAD (72); upper airway infections, including recurrent rhinitis and rhinosinusitis are found in 48–78% of these patients (71–73). However, as mentioned above, not all patients experience this problem and this might be due to a compensatory function of IgM, which has functional similarities to IgA and is often increased in these individuals.

IgA has shown to be very effective at tackling viruses in virus-infected epithelial cells and in redirecting antigens to the lumen when they enter the lamina propria (74). Upper airway infection with certain viruses such as rhinovirus (75), influenza (76), and SARS-CoV2 (77) has shown to induce an increase of S-IgA in the nasal lavage and for influenza, it has been proven that this nasal S-IgA plays an important role in the protection against viral infection in humans (76). This is supported by murine studies where transfer of nasal IgA from immunized to naïve mice leads to protection against influenza infection (78) and mice lacking S-IgA have increased viral load after intranasal challenges (79). No articles are currently discussing the role of IgA in the development of acute bacterial rhinosinusitis, however, patients with cystic fibrosis and CRS who were chronically infected with *Pseudomonas aeruginosa*, showed increased S-IgA concentrations in their nasal secretions (69, 80). At the level of the lower airways it has been shown that reduced respiratory S-IgA production can lead to an increased susceptibility toward *P. aeruginosa* (81). More proof of the importance of S-IgA in the prevention of recurrent upper airway infections is coming from endurance athletes. Strenuous exercise has shown to decrease S-IgA production as measured in the saliva and there is evidence that this decrease is linked to the occurrence of increased upper respiratory tract infections witnessed in elite athletes (82–84).

### Role of IgA in Allergic Rhinitis

Allergic rhinitis (AR) is the most common cause of chronic rhinitis and its prevalence is situated between 23 and 30% in Europe (85). In genetically predisposed atopic individuals, DCs that are exposed to respiratory allergens induce an immunological shift of T-cells toward the Th2 subtype with a production of allergen-specific IgEs by activated B-lymphocytes. After this sensitization phase, re-exposure leads to a crosslinking of the allergen with the mast cell-bound specific IgEs which causes mast cell degranulation and acute allergic symptoms (3). Th2 related cytokines such as IL-4, IL-5, IL-13 attract eosinophils to the nose that release cytotoxic granule proteins and other products (such as lipids and extracellular traps) in the nasal mucosa, giving rise to airway oedema, damage, and remodeling.

Several studies have shown that S-IgA is a potent stimulus for eosinophils and among all immunoglobulins S-IgA even represents the main trigger of eosinophil degranulation, possibly due to eosinophils expressing a specific receptor for SC (86, 87). Blood eosinophils incubated with S-IgA *in vitro* release large amounts of eosinophil cationic protein, eosinophil peroxidase, eosinophil-derived neurotoxin, as well as IL-4 and IL-5 (88, 89). In addition to eosinophils, basophils also degranulate on activation by S-IgA and S-IgA may thus favor a Th2 profile through the modulation of the cytokine response of these cells (90). In AR patients, nasal allergen challenge induces a biphasic increase of IgA in the nasal mucosa (91), similar to IgE, and specific nasal IgA responses have been reported in patients with AR sensitized to house dust mite (92), grass (93), ragweed (94), birch pollen (95), and red cedar (96). In the latter study, IgA levels in nasal lavages were shown to correlate with nasal symptoms (96). Nevertheless, this view of the IgA response as a pathogenic mechanism in AR acting along with IgE, is contested by several observations: first, SIgAD or delayed serum IgA production in childhood is a well-known risk factor for atopy (97, 98) and patients with sIgAD suffer more frequently from AR compared to the general population (71, 72). Second, in contrast to the specific IgE response, there is evidence that the production of allergen-specific IgA seems to play a rather protective role leading to the induction of tolerance toward the respiratory allergens. Healthy individuals show for example allergen-specific IgA antibodies but no IgE, and allergic patients show lower total IgA levels and a relative deficiency in allergen-specific IgA antibodies in their serum compared to healthy controls (13, 14). In neonatal studies, S-IgA in breast milk has been linked to reduced risk for developing atopic diseases (99–102). At a later age, high salivary S-IgA levels were associated with less development of allergic symptoms in a group of sensitized Swedish children (103). Also, a biopsy study performed in adult AR patients showed reduced levels of tissue IgA compared to healthy controls, which was linked to a decreased pIgR expression at the level of the epithelium (15). In addition to these human data, several animal studies support this theory of a protective role of IgA in AR. One study showed that intranasal administration of ragweed-specific IgA protected against allergic inflammation in sensitized mice (104). Another murine study showed that the production of specific IgA in neonatal mice prevented the development of cockroach allergy (105).

More evidence for this tolerance-inducing role of IgA is coming from allergen immunotherapy (AIT) research as will be discussed below. However, the mechanisms by which IgA can prevent or modulate AR still need to be elucidated.

### Role of IgA in Non-allergic, Non-infectious Rhinitis

In about 20–30% of chronic rhinitis patients, no infection nor IgE-mediated allergy can be demonstrated and this pathology is addressed as non-allergic, non-infectious rhinitis or short “NAR” (106). NAR comprises a heterogeneous group of chronic rhinitis subtypes, such as drug-induced rhinitis, hormonal-induced rhinitis and occupational rhinitis. However, in about 50% of the NAR patients, no specific causal factor can be found and this is addressed as idiopathic rhinitis (IR) (107). The disease mechanisms of NAR patients are much less studied than their allergic peers and to our knowledge, no studies have investigated the course nor the role of IgA in NAR.

### Role of IgA in Chronic Rhinosinusitis

Regarding IgA in CRS, it has been reported that 16.7% of patients with CRS have low levels of IgA with 6.2% of them matching the definition of SIgAD (108). Conversely, SIgAD present more frequently with CRS (up to 78%) compared to individuals with normal antibody levels (72).

Several studies have studied the IgA production in CRS patients (Table 1). At the serum level, all studies seem to agree that no significant differences are detected between CRS patients and controls (111, 116). However, at the level of local IgA production, differences are detected between CRS patients and healthy controls. Four studies found higher levels of IgA (total and/or IgA1) in sinus tissue from patients with CRSwNP (15, 109, 113, 115) and three of them showed increased numbers of IgA+ plasma cells (109, 113, 115). Two studies reported also on an overexpression of BAFF, an important inducer of local IgA class switching, in CRSwNP patients (112, 115). These findings indicate an increase in the local IgA production by type 2 inflamed polyp tissue (as is the case for IgE) and most of the plasma cells detected in NP tissue are actually of the IgA producing type (118). One recent article suggests the possibility of local conversion of bacterial antigen-specific IgA to IgE that might occur in a Th2 environment (110), but more insight is needed to confirm its relevance.

**Table 1:** Human studies investigating the role of IgA in CRS.

**References**	**Populations**	**IgA expression in tissue**	**IgA in serum**	**IgA in secretions**	**pIgR expression tissue**
Aazami et al. (109)	36 CRSwNP, 12 CRSsNP, and 22 healthy controls	- Increase of total IgA+ cells in the lamina propia of both CRS groups	Not investigated	Not investigated	Not investigated
		- Increase of total IgA+ cells in the epithelium of patients with CRSwNP			
		- Increase of total IgA and IgA1 in patients with CRSwNP (protein level)			
Takeda et al. (110)	46 CRSwNP and 15 healthy controls	- No significant differences in patients with CRSwNP	Not investigated	Not investigated	Not investigated
		- IgE class switching from IgA-producing B cells in patients with CRSwNP			
Aazami et al. (111)	10 CRSwNP, 10 CRSsNP, and 10 healthy controls	Not investigated	- No significant differences in total IgA levels and subclasses (IgA1, IgA2) in serum among groups	Not investigated	Not investigated
Dilidaer et al. (112)	25 CRSwNP, 12 CRSsNP, and 10 healthy controls	- Increase of BAFF in patients with CRSwNP	Not investigated	Not investigated	Not investigated
Sokoya et al. (113)	6 CRSwNP, 6 CRSsNP, and 6 healthy controls	- Increase of IgA+ cells in patients with CRSwNP	Not investigated	Not investigated	Not investigated
		- Increase of IgA expression in patients with CRSwNP			
Tsybikov et al. (114)	54 CRSwNP, 46 CRSsNP, and 40 healthy controls	Not investigated	Not investigated	- Increased sIgA in both CRS groups, being more pronounced in patients with CRSwNP	Not investigated
Hupin et al. (15)	10 CRSwNP, 13 CRSsNP, and 20 healthy controls	- Decrease of IgA in patients with CRSwNP	Not investigated	- No significant differences among groups	- Decrease of pIgR in patients with CRSwNP
				- Decrease of specific IgA vs *S. aureus* enterotoxin B in patients with CRSwNP	- Decrease of SC concentration in CRSwNP (in nasal secretions)
Kato et al. (115)	60 CRSwNP, 39 CRSsNP, and 30 healthy controls	- Increase of IgA in patients with CRSwNP	Not investigated	- Increase of BAFF in patients with CRSwNP	Not investigated
		- Increase of BAFF in patients with CRSwNP			
		- Increase of BAFF in patients with CRSwNP			
Van Zele et al. (116)	15 CRSwNP, 15 CRSsNP, and 10 healthy controls	- Increase of IgA levels in patients with CRSwNP	- No significant differences in IgA levels among groups	Not investigated	Not investigated
Tan et al. (117)	44 CRSwNP, 25 CRSsNP, and 22 healthy controls	- Increase of total IgA levels in patients with CRSwNP	Not investigated	Not investigated	Not investigated
Chee et al. (108)	78 patients with refractory sinusitis	Not investigated	- Below-normal range IgA levels in 13 of 78 patients	Not investigated	Not investigated
Sánchez-Segura et al. (118)	19 CRSwNP, patients' peripheral blood as control	- Increase of IgA-producing cells in nasal polyp tissue	Not investigated	Not investigated	Not investigated

With regards to S-IgA levels in nasal secretions, results are a bit less straightforward; while a large study performed by Tsybikov et al. (114) found increased S-IgA levels in 54 patients with CRSwNP compared to 40 healthy controls, this increase was not in keeping with the—smaller—study by Hupin et al. (15). Although, they confirmed the previously described increase in tissue IgA in CRSwNP patients, this was not translated into higher S-IgA levels in the nasal secretions, but rather due to an accumulation of IgA in the subepithelial tissue. This could be explained by the fact that CRSwNP patients showed significantly lower expression levels of pIgR on their epithelium, leading to a reduced transepithelial transport of the immunoglobulin. Although, no significant differences in S-IgA were detected, they did demonstrate reduced antigen-specific IgA levels directed toward *S. aureus* Enterotoxin B (SEB) (15), a powerful superantigen linked to the severity of Th2 inflammation. Most of the other studies looking at IgA levels in CRS did not characterize the specificity of IgA in patients. However, other study found elevated mucosal levels of autoreactive IgA to nuclear antigens, including double stranded DNA and basement membrane components that might contribute to the disease mechanism (117).

Very little additional information can be retrieved from animal models, mostly due to the lack of well-established CRS models. Only one article reported on increased S-IgA levels in nasal secretions of mice with experimentally induced CRS (119).

## Role of IgA in Lower Airway Disease

It is well-established that a close link exists between upper and lower airway disease and that inflammation in one part of the airway can influence the homeostasis of the other, a phenomenon that is referred to as “global airway disease” (120). Patients suffering from AR and especially eosinophilic CRS often present with lower airway problems such as asthma, bronchial hyperreactivity, or chronic obstructive pulmonary disease (COPD) and overlap in disease mechanisms is frequently seen.

At the level of IgA research in the lower airways, most of the studies have been performed in COPD. Is has been shown that patients with COPD low serum IgA levels show a higher risk for exacerbations (121). Seminal studies from two groups reported a downregulation of bronchial epithelium pIgR expression in patients with COPD (122–124) which correlated with airflow limitation and neutrophilic infiltration (122, 123). Mechanisms of this defect include a proteolytic degradation of the pIgR by neutrophil-derived proteinases (125) and intrinsically altered signaling pathways in the epithelium mediated by TGF-b1 and Wnt/b-catenin (124, 126). Similar to what is seen in patients with CRS, local IgA synthesis and production by B cells seems is upregulated in lung tissue (127), but does not translate into increased concentration of S-IgA in bronchial secretions due to the defect in pIgR-mediated transepithelial transport.

In addition, one study showed a link between IgA and eosinophils in patients with COPD, with lower eosinophil numbers being related to lower IgA levels in the bronchoalveolar lavage (BAL) fluid and higher abundance of pathogenic bacteria (128). Also, antigen-specific antibody responses were affected as patients with severe COPD showed a defective IgA response against *P. aeruginosa*, which may contribute to chronic/recurrent infections in such patients (129).

The role of IgA in asthma has been less explored. As is the case for AR, SIgAD patients present more frequently with allergic asthma, as well as with allergic sensitization to house dust mite in general, compared to individuals with normal IgA levels. Severe asthma patients present with lower serum IgA levels compared to healthy controls and lower S-IgA levels were negatively correlated with lung function and positively with asthma symptoms (130). A recent study shows significantly lower serum IgA levels in asthmatics with recurrent infections (131). As shown in COPD, IgA levels were correlated to eosinophil activation in previous studies, with correlations between IgA (total and allergen-specific) and eosinophilic cationic protein (ECP) as marker of eosinophil degranulation. This might be due to direct S-IgA-induced eosinophil degranulation (88, 132) since FcαR expression is increased on eosinophils from allergic asthmatics (133) and eosinophils from asthmatic patients do not need additional cytokine-priming to bind IgA *in vitro* (134). Conversely, our group reported a reduced pIgR expression in the bronchial epithelium from patients with asthma, with *in vitro* experiments indicating that IL-4R activation (and thus a type 2 immune environment) may account for this epithelial defect in asthma (68).

A further evidence of the complex interplay between microenvironment and host regulation of epithelial pIgR expression is coming from studies in CF, where our group showed that in the dedifferentiated lower airway epithelium (135), the intrinsic downregulation of pIgR expression seen upon CFTR dysfunction, is upregulated and overcome *in vivo*, likely as a result of the presence of opportunistic bacteria such as *P. aeruginosa* (69). IgA immunity in CF was recently reviewed in details (136).

## Treatments Affecting IgA Biology

When looking at the above-mentioned data, we can conclude that still very little is known on the role and mechanisms of IgA in the development of inflammatory upper airway disease. Nonetheless, they also seem to implicate the immunomodulatory role of this immunoglobulin in AR and CRS. Although strategies aiming at increasing S-IgA levels might have the potential to improve the respiratory microbiome composition, enhance the defense against inhaled pathogens and induce tolerance toward allergens, little therapeutic options are currently available that specifically target the IgA production or its effects. Yet, several well-known therapies for treating chronic inflammatory upper airway disease are known to influence the IgA production and they are listed below.

### IgA Replacement Therapy

Patients presenting with agammaglobulinemia are successfully treated with IgG replacement therapy, which reduces the frequency of invasive bacterial infections. Due to the incapacity of IgA to activate complement by itself, agammaglobulinemia patients do generally not receive IgA replacement therapy, nor do patients with SIgAD. However, agammaglobulinemia patients on IgG treatment often still present with chronic respiratory disease (137–139) and IgA-enriched immunoglobulin preparations could offer a solution. A limited number of these preparations, also containing IgM (Pentaglobin and Trimodulin) have been tested in humans (140). These preparations showed better opsonizing capacities toward certain pathogenic bacteria compared to IgG replacement alone (141, 142) with better clinical outcomes regarding respiratory infections. IgA-only enriched IgG preparations have previously shown successful results in treating intestinal infections in children (143, 144) but have not been tested for the prevention or treatment of respiratory infections. Endonasal administration of serum-derived IgA in children at risk for recurrent airway infections, reduced the number of infectious episodes and increased their endogenous IgA production compared to placebo (145). Although, these results seem promising, the downside of these preparations is that the enrichment is based on serum-derived IgA, which is less resistant to bacterial proteases compared to S-IgA because of the abundance of the IgA1 isoform and the absence of SC.

### Allergen Immunotherapy

Allergen immunotherapy (AIT) is unlike anti-allergic drugs the only disease-modifying treatment option for AR, which has been shown to have long-term benefits (146). AIT is based on the administration of the causal allergens in high dosages in order to modulate the immune response of the allergic patient *via* an immune deviation of Th2-cell response toward a Th1-cell response with the consequent suppression of Th2 cells, induction of regulatory T and B cells and induction of IgG-blocking antibodies (104). Although, we know that both the subcutaneous (SCIT) and sublingual (SLIT) administration routes are very effective in treating AR, several unanswered questions remain regarding its mechanisms. Successful AIT is associated with an increase in IgA responses (100); in a 2-year double blind trial, grass pollen AIT induced a shift in allergen-specific antibody response toward IgA2, which correlated with increased local TGF-β expression and induced monocyte IL-10 expression (147). A recent study comparing subcutaneous and sublingual grass pollen AIT reported mainly increases in allergen specific IgA after sublingual AIT compared to the subcutaneous form, suggesting a more important role of this immunoglobulin in the mechanism of SLIT compared to SCIT (101). It is believed that IgA acts as a scavenger that binds the allergens in the mucosal lumen before they can trigger pro-inflammatory signals by binding their specific mast-cell bound IgE. Data to support this is coming from murine experiments; it has been shown that intranasal treatment of mice with antigen-specific monoclonal IgA antibody prevented increases in airway hyperreactivity, tissue eosinophilia, and IL-4 and IL-5 production after allergen challenge (104), suggesting that neutralization of aeroallergens by IgA is a protective mechanism achieving a form of molecular allergen avoidance.

### Bacterial Lysates

It has been postulated that exposure to bacterial products early in life, reduces the risk of developing allergies and airway infections by increasing the immune response efficacy. Based on this concept, oral bacterial lysates have been tried and proven successfully in the prevention of recurrent viral respiratory tract infections (148–150). The effect is believed to be elicited by the modulation of DC activity, monocyte-macrophages, B-cells, regulatory T cells, and airway epithelial cells (151). One of the studies that showed a beneficial effect of oral bacterial lysate on recurrent airway infections in patients at risk, demonstrated an increase in serum and S-IgA levels, which coincided with the administration of the bacterial lysate (151). More studies are needed to investigate the clinical relevance of this S-IgA increase and its potential consequence on the nasal microbiome.

### Antibiotics

Antibiotics are widely used in the treatment of acute and chronic rhinosinusitis, even in more chronic protocols, in spite of the restricted indications mentioned in the European guidelines (5). The use of antibiotics is known to affect the intestinal and respiratory microbiota, possibly leading to an imbalance of their microbiome by over- or under-growth of certain species. One study combining both murine and human data showed that antibiotic use led to a microbial shift that reduced the TLR- and APRIL-dependent respiratory S-IgA production, leading to an increased susceptibility toward certain pathogens such as *P. aeruginosa*. In mice, the nasal application of polyclonal intestinal IgA attenuated the severity of *P. aeruginosa* infection and its mortality, after an antibiotic-induced decrease in IgA (81).

## Future Perspectives

### Boosting the Endogenous IgA Production

Several studies mentioned above have indicated that a rich microbial environment can contribute to mucosal tolerance and protective IgA responses, leading to an improved protection against respiratory infections and inflammation. This has brought forward the idea of using a microbial-derived molecule as a potential candidate to stimulate protective IgA responses and preventing the development of infections and allergy. Cholera toxin is the most widely experimentally used mucosal adjuvant, which is capable of inducing an IgA response through TLR-primed DCs. Studies in mice have shown that the co-administration of an experimental allergen and cholera-toxin prevents sensitization to the allergen (152, 153). Others found that intratracheal administration of cholera toxin can suppress allergic inflammation through the induction of airway luminal IgA secretions, an effect that was not seen in mice lacking S-IgA (154). This has led to the hypothesis that the administration of cholera toxin to patients with impaired IgA synthesis could contribute to a broader antibody repertoire with increased mucosal IgA levels leading to an improved mucosal immunity and local homeostasis (155). Nonetheless, this remains to be further explored in human disease.

### S-IgA Replacement Therapy

In comparison to serum IgA, S-IgA is much less sensitive to proteolytic activity of bacteria and the SC contributes to the efficiency of its immune exclusion capacity (47). Therefore, local replacement therapy of S-IgA might be more effective in patients suffering from agammaglobulinemia, SIgAD and other immune deficiencies, at preventing recurrent infections compared to replacement therapy using serum IgA. Anecdotal studies in mice have suggested that local application of S-IgA can be protective against certain infections (81, 156) and this is a potential track to be explored for treating refractory or recurrent infections.

### Passive Allergen Immunotherapy With Allergen-Specific IgA

Although AIT has shown to provide long-lasting beneficial effects for a substantial number of AR patients, SCIT still carries a risk of important adverse reactions and SLIT is susceptible to failure due to therapeutic compliance issues. Also, it shows variable efficacy between patients, and generally takes 3–5 years to induce tolerance. For these reasons, researchers have been looking for alternative ways to induce allergen tolerance. One of the possibilities is the administration of a so-called passive AIT under the form of antigen-specific immunosuppressive antibodies that have the ability of competing with IgE for allergen binding and functionally preventing the immediate hypersensitivity response. This hypothesis has been tested in both cat- and birch-allergic patients, receiving subcutaneous injections with a pre-selected allergen-blocking IgG against the respective major allergens showing beneficial results for over 2 months after only one injection (157, 158). Because of the mucosal advantage of IgA over IgG, a similar therapeutic potential may lie in the local administration of isolated allergen-specific IgA, which has been shown to be effective in an *in vivo* mouse model of airway allergy (104). Nonethesless, the efficacy and applicability of this approach in allergic patients needs however to be studied.

## Conclusion

The role of IgA in upper airway homeostasis and disease is complex and remains controversial. This is probably due to its dual role combining both suppression and activation of the local immune response depending on the environmental context. Although, this immunoglobulin has shown to play a major role in developing tolerance against allergens and beneficial microbes in the gut, it remains largely understudied in the airways. Several studies however, indicate similar pathways in the field of respiratory allergies, infections and chronic inflammation. For this reason, more studies investigating its role and its potentially disease-modifying actions are needed in order to invest in potential therapeutic targets that interfere with its production.

## Author Contributions

AS: drafting, writing, and revising manuscript. SG and PR: writing and revising manuscript. CP: conceptualizing, writing, and revising manuscript. VH: conceptualizing, drafting, writing, and revising manuscript. All authors contributed to the article and approved the submitted version.

## Funding

SG, VH, and CP are postdoctoral specialists of the Fonds National de la Recherche Scientifique (grants 1R60121F, 1R20221F, and 1R01618, respectively), Belgium.

## Conflict of Interest

The authors declare that the research was conducted in the absence of any commercial or financial relationships that could be construed as a potential conflict of interest.

## Publisher's Note

All claims expressed in this article are solely those of the authors and do not necessarily represent those of their affiliated organizations, or those of the publisher, the editors and the reviewers. Any product that may be evaluated in this article, or claim that may be made by its manufacturer, is not guaranteed or endorsed by the publisher.
